# 

**Published:** 1904-01-15

**Authors:** 


					﻿THE
DENTAL REGISTER

EDITED BY
NELVILLE S. HOFF, D. D. S.,
ANN ARBOR, MICH.
—.	—	-—	.	----------- - L
Vol.iLsVMJ. i D^ANUiARY 15, 1904.	No. 1.
PRICE, ONE DOLLAR A YEAR IN ADVANCE.
PUBLISHED MONTHLY BY
SAMUEL A. CROCKER & CO.,
THE OHIO DENTAL AND SURGICAL DEPOT.
tEZS to 31) W. "tli St., Ciiieiiuiiiti, O.
Entered at the Postoffice at Cincinnati, 0., as second-class mail matter.
				

